# The influence of aging on the number of neurons and levels of non-phosporylated neurofilament proteins in the central auditory system of rats

**DOI:** 10.3389/fnagi.2015.00027

**Published:** 2015-03-11

**Authors:** Jana Burianová, Ladislav Ouda, Josef Syka

**Affiliations:** Department of Auditory Neuroscience, Institute of Experimental Medicine of the Czech Academy of Sciences, PragueCzech Republic

**Keywords:** SMI-32, neurofilaments, number of neurons, aging, auditory system

## Abstract

In the present study, an unbiased stereological method was used to determine the number of all neurons in Nissl stained sections of the inferior colliculus (IC), medial geniculate body (MGB), and auditory cortex (AC) in rats (strains Long Evans and Fischer 344) and their changes with aging. In addition, using the optical fractionator and western blot technique, we also evaluated the number of SMI-32-immunoreactive (-ir) neurons and levels of non-phosphorylated neurofilament proteins in the IC, MGB, AC, and visual cortex of young and old rats of the two strains. The SMI-32 positive neuronal population comprises about 10% of all neurons in the rat IC, MGB, and AC and represents a prevalent population of large neurons with highly myelinated and projecting processes. In both Long Evans and Fischer 344 rats, the total number of neurons in the IC was roughly similar to that in the AC. With aging, we found a rather mild and statistically non-significant decline in the total number of neurons in all three analyzed auditory regions in both rat strains. In contrast to this, the absolute number of SMI-32-ir neurons in both Long Evans and Fischer 344 rats significantly decreased with aging in all the examined structures. The western blot technique also revealed a significant age-related decline in the levels of non-phosphorylated neurofilaments in the auditory brain structures, 30–35%. Our results demonstrate that presbycusis in rats is not likely to be primarily associated with changes in the total number of neurons. On the other hand, the pronounced age-related decline in the number of neurons containing non-phosphorylated neurofilaments as well as their protein levels in the central auditory system may contribute to age-related deterioration of hearing function.

## Introduction

One intriguing feature of the naturally aging mammalian brain is its relatively slow rate of neuronal loss. Initial studies that used biased microscopic techniques rather overestimated its extent (Brody, 1955; Devaney and Johnson, 1980) while modern quantitative methods have adjusted these estimates, rendering the loss less dramatic, and not exceeding approximately 10% in the case of human cortex (Pakkenberg and Gundersen, 1997). More detailed research in rodents have revealed variability across brain structures, but generally the decline in the number of neurons has been found to be marginal, falling below statistical significance in several brain areas, including the hippocampus (Rapp and Gallagher, 1996) and entorhinal cortex (Merrill et al., 2001). Therefore, naturally deteriorating brain function represented by cognitive processes cannot be simply accounted for by a numerical decline in the neuronal population.

Aging of the central auditory system has been intensively studied in an attempt to reveal the mechanisms underlying gradual age-related hearing loss (ARHL), or presbycusis. While peripheral alterations are relatively well documented (Schuknecht and Gacek, 1993; Gates and Mills, 2005; Buckiova et al., 2007) the central part of presbycusis remains far from being elucidated (for review see Syka, 2002; Caspary et al., 2008; Canlon et al., 2010) including the question of whether it develops independently or as a consequence of the restricted peripheral input. Studies in rodents have suggested that central presbycusis may result from disturbances of the inhibitory function mediated by GABAergic neurons in higher parts of the auditory pathway (Caspary et al., 2008). These studies demonstrated an age-related reduction in levels of mRNA and proteins or reduced immunoreactivity for glutamate decarboxylase (Ling et al., 2005; Burianova et al., 2009), a key GABA-synthesizing enzyme. In addition, the reduction was also present in calcium binding proteins such as parvalbumin, calbindin, and calretinin (Ouda et al., 2008, 2012a) which are prevalently found in the GABAergic neurons (Kubota et al., 1994; Gonchar and Burkhalter, 1997; Jinno and Kosaka, 2006; Gonchar et al., 2007). This is particularly important given the fact that GABAergic neurons play a crucial role during the processing of temporal parameters of complex sounds (Kawaguchi and Kubota, 1998; Bartos et al., 2007). Indeed, elderly people experience an inability to comprehend speech in a noisy environment (Pronk et al., 2013) and old rats display deficits in the detection and discrimination of gaps in a continuous noise (Suta et al., 2011).

There are other markers of aging in the central auditory system that reflect disturbances of its function. Non-phosphorylated epitopes of heavy and medium neurofilament subunits which are recognized by SMI-32 antibodies (Sternberger and Sternberger, 1983) could be such a candidate molecule. Together with a light subunit, these subunits form a neurofilament triplet thought to be associated with the level of myelination and the fast conduction of axons. Although SMI-32-ir neurons usually constitute a minority of the neuronal population in a particular central auditory brain structure (Ouda et al., 2012b), their conductance properties may play an important role in carrying and processing fast auditory signals. In the cerebral cortex, SMI-32-ir neurons are more abundant in layer V, often as subcortically projecting pyramidal neurons characterized by long apical dendrites (Ouda et al., 2012b) and bursts of action potentials (Voelker et al., 2004). SMI-32-ir neurons in the neocortex have been shown to be particularly vulnerable during Alzheimer’s (Hof and Morrison, 1990) and Huntington (Cudkowicz and Kowall, 1990) disease, raising concerns over whether they are also poorly resistant during natural aging. These neurons have not yet been evaluated in the central auditory system, but related studies have confirmed age-related alterations in SMI-32 recognized epitope, indicating a progressive hyperphosphorylation of the neurofilaments (Vickers et al., 1992; Veeranna et al., 2011).

The main aim of the present study was originally to evaluate changes in SMI-32 immunoreactivity during natural aging in upper parts of the central auditory system, i.e., the inferior colliculus (IC), medial geniculate body (MGB), and auditory cortex (AC). However, these changes cannot be fully understood and properly interpreted without knowing whether there are age-related changes in the total number of neurons in each of the studied structures. Reliable knowledge of the total number of neurons in the different regions of the auditory system and their respective changes with aging can aid us in understanding the principles of presbycusis. To date, only a few unbiased assessments have been published with the aim of determining the total number of neurons in the nuclei and regions of the central auditory system of rats (Kulesza et al., 2002; Ouda et al., 2012b). Thus, the second aim of this study is to provide an unbiased estimate of the neuron numbers.

We employed an approach used in our previous work, comparing age-related changes occurring in Fischer 344 strain, known for its accelerated aging, including early onset of hearing loss, with Long Evans rats, which has preserved hearing function until late senescence.

## Materials and Methods

### Animals

In total, 22 Long Evans rats (14 young animals, 3–6 months old, and 8 aged animals, 29–35 months old), and 16 Fischer 344 rats (8 young animals, 3–4 months old, and 8 aged animals, 20–25 months old) were used in the experiments. Ages of the aged animals were intentionally chosen to be the highest possible, derived from the expected life span of the correspondent rat strain. All Long Evans rats were obtained from our local facility at the age of 2 months and reared in-house under the same conditions. All Fischer 344 rats were purchased at 2 months of age from Charles River Deutschland (Charles River Wiga GmbH, Sulzfeld, Germany) and then reared in-house under standard rearing conditions. All animals were housed in age-matched groups of two or three per cage under standard laboratory conditions in a constant environment and a 12/12 h normal light/dark cycle; food and water remained available *ad libitum*. We did not observe signs of any middle ear infection in any animal during their stay in our animal facility. The care and use of animals and all experimental procedures followed the principles of laboratory animal care and were performed in compliance with the guidelines of the Ethical Committee, Institute of Experimental Medicine of the Czech Academy of Sciences, and the Declaration of Helsinki.

### Nissl Staining

#### Animals

Fourteen male Long Evans rats (10 young animals, 3–6 months old, and 4 aged animals, 29–33 months old) and 8 Fischer 344 rats (4 young animals, 3 months old, and 4 aged animals, 20–24 months old) were used in the experiments. Results obtained from the study of the young Long Evans rats have already been published (Ouda et al., 2012b).

#### Tissue Processing

Under deep anesthesia (ketamine 50 mg/kg + xylazine 8 mg/kg, *i.m.*) the animals were transcardially perfused with saline followed by 4% paraformaldehyde fixative in 0.1 M phosphate buffer (pH 7.4). Following 15 min of perfusion, the brains were removed, postfixed overnight at 4°C (same fixative), and then cryoprotected with 10, 20, and 30% sucrose in phosphate buffer for 1 day each.

#### Nissl Staining

**FIGURE 1:**
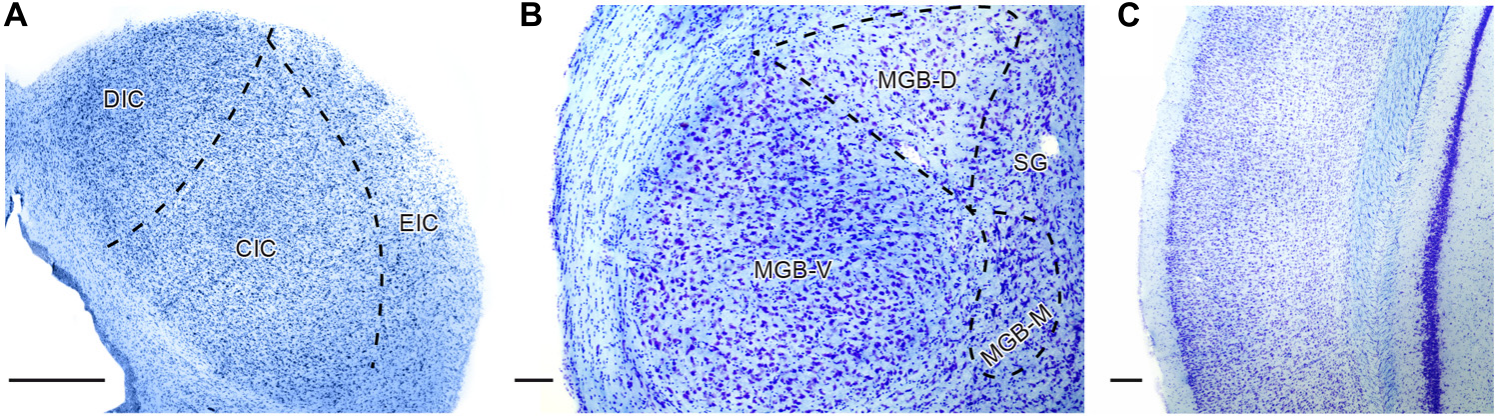
**Illustration of Nissl staining in the inferior colliculus (IC; **A**), medial geniculate body (MGB; **B**), and auditory cortex (AC; **C**) of young Long Evans rat with delineation of the principle subdivisions in the particular structure.** DIC, dorsal cortex of the IC; CIC, central nucleus of the IC; EIC, external cortex of the IC; MGB-V, -D, -M – ventral, dorsal, medial divisions of the MGB; SG, suprageniculate nucleus of the MGB. Scale bar **(A,C)** = 200 μm, **(B)** = 100 μm.

Coronal serial sections (40 μm thick) were cut with a freezing microtome. Sections were mounted in serial order onto glass slides, air-dried, stained for Nissl substance using standard laboratory protocol with 1% cresyl violet and coverslipped. An illustration of the Nissl stained sections is provided in **Figure 1**.

### Immunohistochemistry – SMI-32

Ten male Long Evans rats (6 young animals, 3–6 months old, and 4 aged animals, 29–33 months old) and 8 Fischer 344 rats (4 young animals, 3 months old, and 4 aged animals, 20–24 months old) were used. The results obtained from the young Long Evans rats have already been published (Ouda et al., 2012b). The tissue was processed as in 2.2.2. coronal serial sections (40 μm thick) were cut with a freezing microtome. Free-floating sections were preincubated in a blocking solution (5% low fat milk in PBS, 1 h) and then immersed in PBS containing the mouse monoclonal antibody SMI-32 against non-phosphorylated heavy and medium neurofilament subunits (1:1000, Covance Research Products, USA) for 18 h (4°C). Sections were incubated with a goat anti-mouse biotinylated secondary antibody (1:200, Sigma-Aldrich) for 1 h, and then with avidin-biotin-peroxidase complex (1:100, Vector Laboratories) for 1 h at room temperature. The reaction was visualized with 0.02% diaminobenzidine (DAB) and 0.01% hydrogen peroxide (15 min). Finally, the sections were mounted on slides, dehydrated and coverslipped.

### Microscopy, Imaging and Stereological Quantification

The number of Nissl stained neurons was evaluated in all sections containing the IC, MGB, and AC, and delineated according to anatomical atlases (Zilles, 1985; Paxinos and Watson, 1998). In addition, the number of SMI-32-ir stained neurons was also evaluated in the rat visual cortex (VC).

In the rat brain, sections for the IC were sampled from bregma -8.0 to -9.2 mm, for the MGB from bregma -5.0 to -6.2 mm, for the AC from bregma -4.2 mm to -5.8 mm, and for the VC from bregma – 4.5 mm to – 9.2 mm (Zilles, 1985).

Estimates of the total number of neurons were inferred from the sample counts of Nissl positive nuclei. For this purpose an unbiased stereological method, the optical fractionator, was used. Measurements were performed using bright-field microscopy (Leica DMRXA microscope) equipped with an X-Y-Z motor stage and Stereo Investigator software (MicroBrightField, Inc., Colchester, VT, USA) was used to perform the optical dissector and nucleator protocol (West et al., 1991; Mayhew and Gundersen, 1996; Andersen and Gundersen, 1999).

First, we randomly selected a section containing the analyzed auditory structure, outlined the selected area of interest (5 × or 40 × objective lens) and then uniformly sampled it. The stereological probes were used in conjunction with a 100 × objective lens, while the counting frames used to obtain the sampling remained the same size in all the examined regions (2500 μm^2^ – 50 × 50 μm) and were formed by two inclusion lines and two exclusion lines. The frequency of the samples depended on the size of the structure (section fraction). While the original thickness of the sections was 40 μm, due to the tissue processing, the average thickness was reduced to approximately 20 μm. The height of the optical dissector was held constant at 10 μm and the first and last 5 μm thicknesses of the section were omitted from the analysis (guard zones). Each neuron in the counting frame was counted when its nucleus came into maximum focus. To exclude glia cells from the analysis we identified them on the basis of their limited or non-visible cytoplasm and small dark nuclei.

The neuronal estimate for a given structure (Nest) was calculated as Nest = number of counted cells × area fraction × section fraction × dissector fraction.

The method of estimating the number of SMI-32-ir neurons was essentially the same as that used in the evaluation of the Nissl stained neurons. Specific differences are described elsewhere (Ouda et al., 2012b).

### Western Blot Protein Analysis

Eight male Long Evans rats (4 young animals, 3–6 months old, and 4 aged animals, 30–35 months old) and 8 Fischer 344 rats (4 young animals, 4 months old, and 4 aged animals, 21–25 months old) were used in the experiments. Anesthetized rats (ketamine 35 mg/kg + xylazine 6 mg/kg, i.m.) were decapitated, their brains quickly extracted and rinsed in ice-cold physiological solution, and the ICs, ACs, and VCs were rapidly removed bilaterally. The samples included the whole IC, the Te1 + Te3 (Zilles, 1985) areas of the AC, and the V1 and V2 areas of the VC (Paxinos and Watson, 1998). All samples were immediately put into dry ice after extraction and stored frozen at -80°C until processed. For the analysis, ICs, ACs, and VCs were homogenized by a Potter–Elvehjem homogenizer in 0.05 M Tris–NaCl (pH 7.4) buffer with protease inhibitors (Sigma-Aldrich). The homogenate was centrifuged at 10,000*g* for 10 min at 4°C. To ensure similar protein loading, the total protein concentration of these extracts was determined using the Bradford method with BSA as the standard (Bradford, 1976). An absorbance of 595 nm was used. Samples (cytosolic fractions) were incubated in boiling water for 10 min at 80°C in sodium dodecyl sulfate–polyacrylamide gel electrophoresis buffer containing 10% glycerol, 2% SDS, 0.05% bromphenol blue and 4 M dithiotreitol. Samples were then subjected to Tris/Tricine/ SDS–PAGE on a 3% bis-acrylamide polyacrylamide gel at 30 mA/gel for 150 min on a Mini-Protean II apparatus (Bio-Rad; Ogita and Markert, 1979). After electrophoresis, the resolved proteins were transferred (Bio-Rad Mini Protean II transblot apparatus at 350 mA for 60 min at 4°C in 25 mM Tris, 192 mM glycine, 20% methanol, 0.1% SDS) to a nitrocellulose membrane (Amersham, Biosciences; Towbin et al., 1979). Equal loading and transfer of the western blot samples were further verified by reversible total protein staining of the nitrocellulose membrane with Ponceau-S reversible membrane staining. Loading control was made using the same amount of samples separated by SDS-PAGE in the same conditions and stained with sensitive Coomassie Blue. Membranes were incubated in 3% non-fat dry milk, in 10% Tris-buffered saline with 0.05% Tween 20 (TBST) for 65 min at room temperature to block non-specific protein binding. After being washed in TBST buffer (three times quickly, 3 × 5 min each), the membranes were probed overnight at 4°C with SMI-32-specific (mouse monoclonal, Covance Research Products, USA, 1:500 in TBST) or actin-specific (mouse monoclonal, Chemicon-Millipore, 1:3000 in TBST) primary antisera. The membranes were washed again and incubated with goat anti-mouse IgG antibody conjugated with horse radish peroxidase (Upstate, 1:7500 in TBST) for 2 h at room temperature. Before enhanced chemiluminescence (ECL), the membranes were washed as described above and stored in TBST for at least 2 h. For ECL, substrates A (Luminol solution) and B (H_2_O_2_ solution) were prepared, mixed 40:1 (Amersham Biosciences) just before use, and poured onto the membranes. The specific signals were detected on autoradiographic film (Kodak MXB). Films were developed at room temperature in a dark room, stopped, fixed, washed under running cold water, and air-dried. Scanning (Canon CanoScan 9000F) and ImageQuant software were used for quantifying the relative abundance of SMI-32 and actin in the individual samples. The amount of protein applied to the gel intentionally varied for each fraction in order to achieve linearity with the intensity × area (volume) of the band on the western blot. Samples from the compared experimental groups were run on the same gel and quantified on the same membrane. To ensure the specificity of SMI-32 and actin immunoreactive proteins, prestained molecular weight protein standards were used (Invitrogen). The levels of SMI-32 were calculated as the ratio of the optical density of the antibody of interest to the optical density of the antibody directed against actin.

### Statistical Analysis

The statistical significance of the differences between the groups was assessed by Mann–Whitney *U* test with the use of GraphPad Prism software (version 4.0). *P*-values of 0.05 or less were considered statistically significant.

## Results

### Long Evans Rats

**FIGURE 2:**
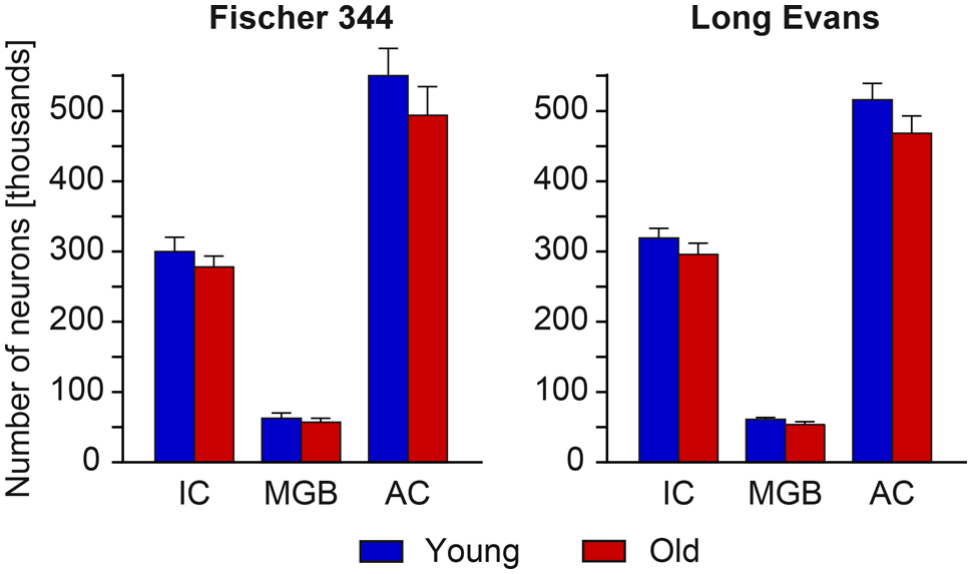
**Numerical estimates of the total number of neurons in the IC, MGB, and AC of young and old rats.** With aging, we found only a mild and non-significant decline in the total number of neurons in all three analyzed auditory structures in both rat strains.

For Long Evans rats, the total numbers of neurons in the particular regions of the auditory pathway in young animals were previously evaluated by Ouda et al. (2012b) and the data obtained from the IC, MGB, and AC serve as the statistical comparison in the present paper. The observed age-related changes in the total number of neurons in all three structures were non-significant. In aged rats, the total number of neurons decreased by 7% in the IC, by 12% in the MGB and by 9% in the AC (**Figure 2**).

**FIGURE 3:**
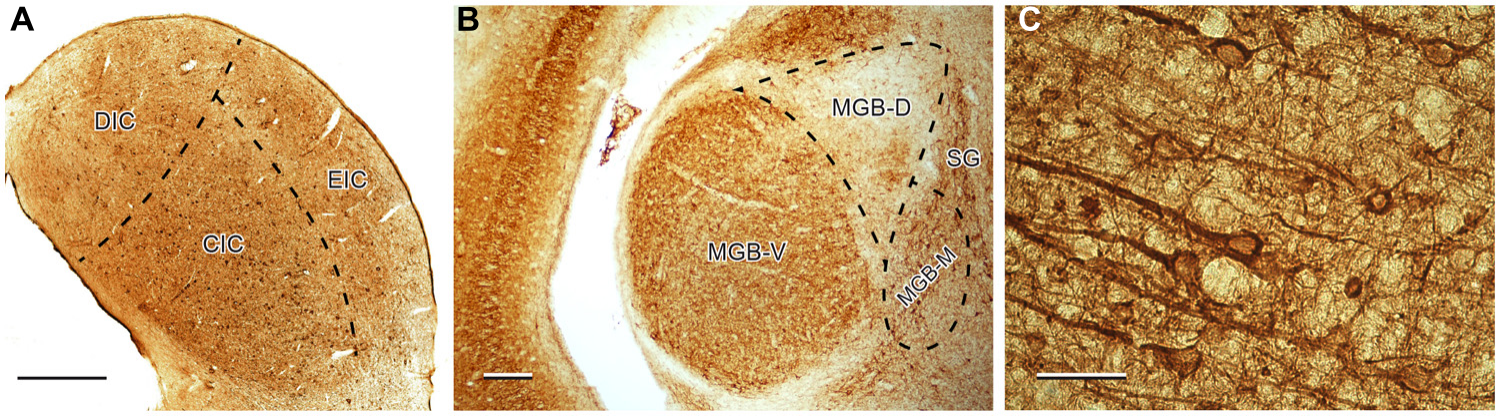
**Illustration of SMI-32 immunoreactivity in the IC **(A)**, MGB **(B)**, and in the Te1 auditory cortical area **(C)** in young Long Evans rats.** DIC, dorsal cortex of the IC; CIC, central nucleus of the IC; EIC, external cortex of the IC; MGB-V, -D, -M, ventral, dorsal, medial divisions of the MGB; SG, suprageniculate nucleus of the MGB. Scale bar **(A,B)** = 200 μm, **(C)** = 50 μm.

The distribution and number of SMI-32-ir neurons was also previously evaluated in Ouda et al. (2012b) and serves as the statistical comparison in the present paper. In both, young and old Long Evans rats, SMI-32-ir neurons were distributed relatively homogenously throughout the whole IC with higher density in the ventral and medial parts of the central nucleus of the IC, i.e., in the high-frequency region, which corresponds to a higher density of neurons apparent in the Nissl staining of this region. In the MGB, the SMI-32-ir neurons were much more prevalent in the ventral division and in the adjacent region of the dorsal division, while the dorsolateral parts of the dorsal division contained only a few positive cells. In the AC, SMI-32-ir neurons were scattered throughout cortical layers II–VI with a dominant position of intensely stained SMI-32-ir pyramidal neurons with thick immunopositive apical dendrites in layers III and V and a more heterogeneous population in layer VI (**Figure 3**).

**FIGURE 4:**
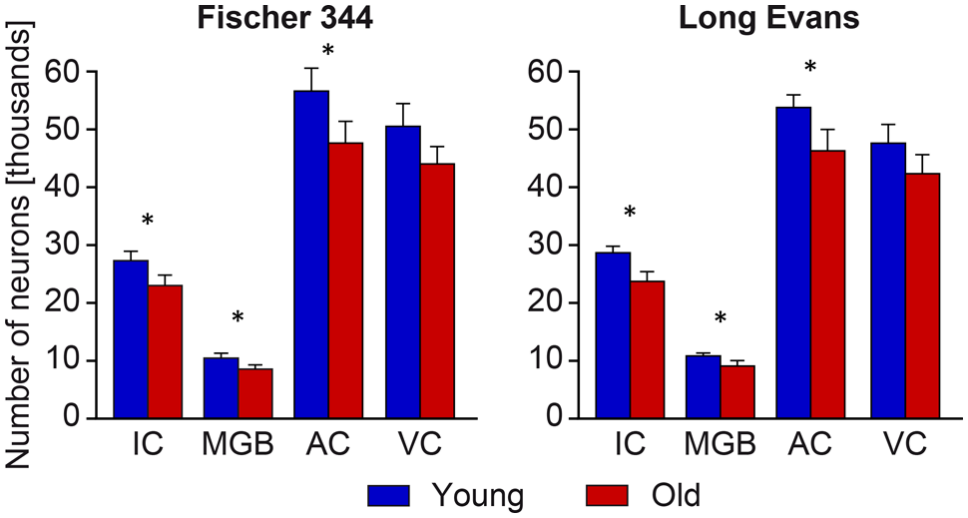
**Number of SMI-32-immunoreactive neurons in the IC, MGB, AC areas (AC – Te1 and Te3) and visual cortex (VC) in young and old rats of both the Long Evans and Fischer 344 rat strains.** The error bars represent SEM (^∗^*P* < 0.05).

**FIGURE 5:**
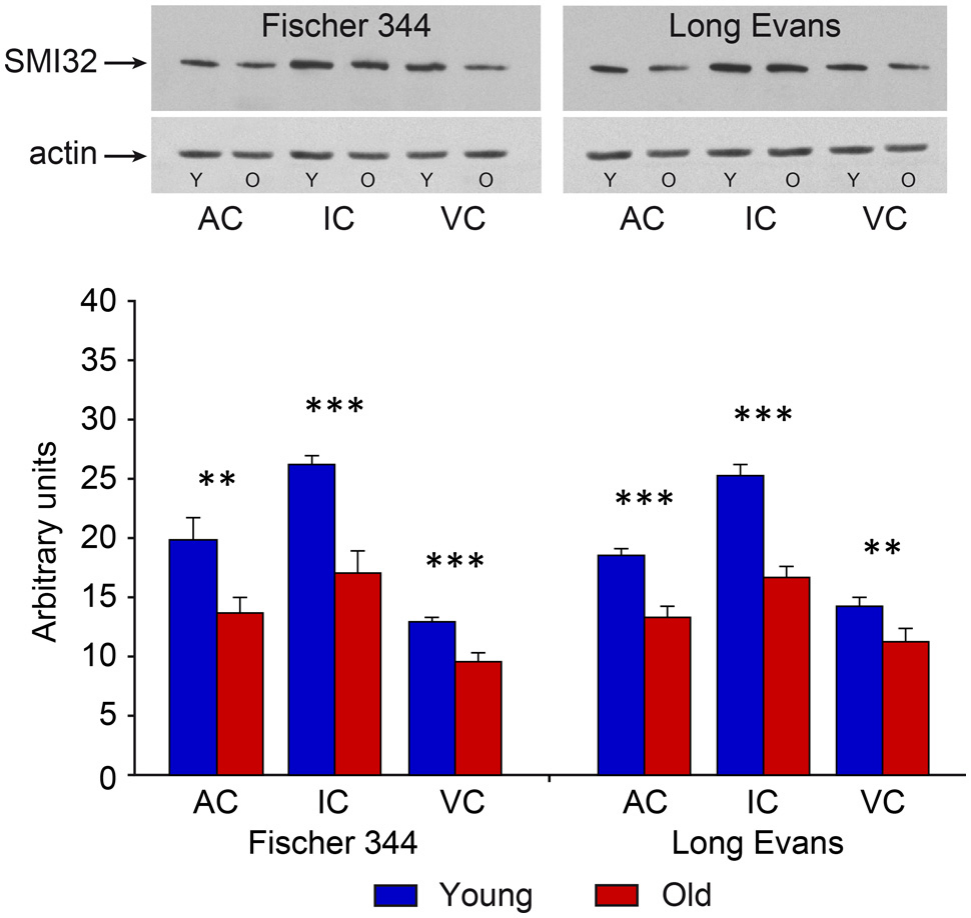
**Results of western blot SMI-32 analysis in young and old animals of both examined strains.** Corresponding representative films of western blot analysis of SMI-32 (200 kDa) developed with the enhanced chemiluminescence (ECL) method. Actin, used as an internal control, was detected at the position corresponding to a molecular weight of 42 kDa. The abbreviations are: AC, auditory cortex; IC, inferior colliculus; VC, visual cortex, Y, young; O, old animals. Arbitrary units are calculated as the ratio of the optical densities of the examined protein and actin (in scanned films analyzed using ImageQuant software). The error bars represent SD (^∗∗^*P* < 0.01, ^∗∗∗^*P* < 0.001).

In contrast with the mild changes in the total number of neurons, the age-related decline in the number of SMI-32-ir neurons was significant in all three examined structures. In the IC the number decreased by 17%, in the MGB by 18% and in the AC by 14% (all *P* < 0.05). In comparison with the AC, the age-related decrease was milder in the VC, 11%, and was not significant. (**Figure 4**). Western blot analysis revealed a significant age-related decline in the levels of non-phosphorylated neurofilaments (SMI-32 positive) in old Long Evans rats in comparison with young animals by 34% in the IC, 28% in the AC, and 21% in the VC (IC and AC *P* < 0.001; VC *P* < 0.01; **Figure 5**).

### Fischer 344 Rats

In young Fischer 344 rats, the observed total numbers of neurons in the IC, MGB, and AC were similar to that found in young Long Evans rats. In addition to this, the observed age-related changes in the total number of neurons in all three structures were also relatively small and non-significant. In aged Fischer 344 rats, the total number of neurons decreased by 9% in the IC, by 11% in the MGB, and by 10% in the AC (**Figure 2**).

The distribution and quantity of SMI-32-ir neurons in the IC, MGB, and AC of young Fischer 344 rats corresponded with the situation present in young Long Evans rats. Also, as in old Long Evans rats, the number of SMI-32-ir neurons decreased significantly in Fischer 344 rats with aging. In the IC, their number decreased by 16%, in the MGB by 20%, and in the AC by 17% (all *P* < 0.05), while in the VC the decrease was lower, 13%, and was not significant (**Figure 4**). Western blot analysis revealed a significant age-related decline in the levels of non-phosphorylated neurofilaments (SMI-32 positive) in old Fischer 344 rats in comparison with young animals by 35% in the IC, 32% in the AC, and 25% in the VC (IC and VC *P* < 0.001; AC *P* < 0.01; **Figure 5**).

## Discussion

### Age-Related Cell Loss in the Examined Auditory Structures

We demonstrated a non-significant age-related decline in both Long Evans and Fischer 344 rats in the IC, MGB, and AC. Our results fully support the view that natural age-related neuronal loss is much less prominent than previously thought. Biased non-stereological methods inferring neuron counts from neuron densities rendered estimates of the age-related neuronal loss in the human VC as high as nearly 50% (Devaney and Johnson, 1980). Later, stereological tools such as the optical dissector enabled the correction of these estimates so that the number of neurons in the whole human neocortex was believed not to reduce by more than 10% with aging (Pakkenberg and Gundersen, 1997). Due to fewer methodological constraints, more reports are available from rodent studies. Although the cerebellum (Rutten et al., 2007) as well as the prefrontal (Stranahan et al., 2012) and occipital cortices (Yates et al., 2008) have been reported to undergo mild neuronal loss, there are multiple brain regions that are resistant to significant age-related neuronal death. These include the hippocampus (Rapp and Gallagher, 1996; Rutten et al., 2007) and entorhinal cortices (Merrill et al., 2001) in which it has been convincingly demonstrated that a natural decline in function (cognitive capabilities) showed no correlation with the number of neurons of its neural substrate (Rapp and Gallagher, 1996; Merrill et al., 2001). Our data suggests that such a decline of function, not associated with neuronal loss, holds true also for the auditory system. Although we did not directly correlate cell numbers of the IC, MGB, and AC with any auditory functions in the present study, we can refer to previous work comparing corresponding cohorts of Long Evans rats. Aged animals of this strain were found impaired in auditory cortex-dependent temporal processing of auditory stimuli (Suta et al., 2011) as well as in responses to startle stimuli (Rybalko et al., 2012), a process thought to involve (among others) the IC.

The survival of neurons in the auditory pathway seems to follow the rule that regions proximal to the periphery are more vulnerable to cell death due to the negative impact of age-related degradation of the cochlea, while structures located more centrally are less affected. Accordingly, aged C57/BL/6J mice, which exhibit fast deterioration of cochlear function, display significant neuronal decline in the dorsal and posteroventral cochlear nuclei (Idrizbegovic et al., 2004). On the contrary, CBA/CaJ mice that have a more preserved cochlear function show only moderate loss of neurons in the dorsal cochlear nucleus (Idrizbegovic et al., 2001). Our current data apparently matches the above mentioned rule as we report relatively preserved neuronal populations in the upper parts of the auditory pathway. Importantly, this holds true also in Fischer 344 rats, i.e., the strain with cochlea degeneration in early senescence. Furthermore, results of our examination of the IC are in agreement with the previous (albeit not unbiased) neuronal estimates, reporting no significant loss in the whole IC of aged C57/BL/6J and CBA mice (Willott et al., 1994), and the central nucleus of the IC in F344 rats (Helfert et al., 1999). The finding that the rat AC does not display substantial neuronal decline is in line with recent data performed on the Long Evans strain by Ouellet and de Villers-Sidani (2014). These authors investigated the temporal trajectory of various neuron subpopulations and found that the total counts of neurons as revealed by Nissl staining showed a remarkable decline in number from P9 to P20, however, following this it slowed down and remained non-significant until the latest age studied (25 months). Thus, our data suggests an extension of this period up until 33 months. On the other hand, Martin del Campo et al. (2012) reported a subtle yet significant loss of neurons of C57/BL/6J mice in the deep (V–VI) cortical layers while the neuron number in the superficial layers remained preserved, suggesting that auditory cortex in the mice might be more vulnerable to neuronal loss than in the rats.

### Age-Related Changes in the Central Auditory System: Involvement of Inhibitory Neurons

Our current data may also enhance the interpretation of previous studies comparing both strains of rats, focusing on the investigation of age-related changes in markers of the inhibitory system (Ouda et al., 2008, 2012a; Burianova et al., 2009). It should be noted here that statistical differences presented in Burianova et al. (2009) were further strengthened by adding more animals of both strains (Supplementary Figure S1). The significant age-related alterations in the markers of inhibitory system found in the cited studies are therefore not accompanied with substantial loss of neurons. We suggest that a diminished expression of proteins in the originally immuno-reactive neurons may be behind the age-related reduction in the detectable numbers of immuno-reactive neurons in the higher regions of the auditory pathway. Such a process could also explain the observed down-regulation of the number of GAD (Burianova et al., 2009) or calcium binding protein expressing neurons (Ouda et al., 2008, 2012a) reported in aged rats. Similarly, the up-regulation of expression or *de novo* expression of proteins in the previously non-expressing neurons, such as the increase in the number of PV-ir neurons in the primary AC of aged rats after intense auditory training (de Villers-Sidani et al., 2010) or the increase in the number of CR-ir neurons in the IC of old CBA mice (Zettel et al., 1997), also supports this hypothesis. The work of Ouellet and de Villers-Sidani (2014) clearly demonstrates how age-related changes in the number of inhibitory cells labeled by a particular marker have their own pattern regardless of the total number of neurons. Further studies, aimed at identifying the rate of particular population cell loss and diminished (or increased) expression of the corresponding marker protein would provide valuable insight into the mechanisms of inhibition disturbance, a putative cause of central presbycusis.

### Age-Related Loss of SMI-32-ir Neurons and Protein Levels

Immuno-reactivity for non-phosphorylated epitopes of heavy and medium neurofilament chains decreased in senescent rats of both strains to a similar degree in the IC, MGB, and AC. The decline was more robust when we evaluated the protein levels using western blot. The results are in accord with the study by Liu et al. (2013) that were conducted on C57BL/6 mice and found an almost three times lower level of protein labeled by SMI-32 in the neocortex of 12 months old mice compared to 5 months old mice. We further found a significant loss in the number of SMI-32-ir neurons (∼15–20%) in each examined structure. Since SMI-32-ir neurons only represent around 10–20% of the IC, MGB, or AC neurons (Ouda et al., 2012b), this statistically significant decline is not contradictory to the non-significant loss of the total number of neurons and may indicate a selective vulnerability of SMI-32-ir neurons to cell death. Indeed, such selective vulnerability has been documented in pathological states such as Alzheimer’s (Hof and Morrison, 1990) and Huntington disease (Cudkowicz and Kowall, 1990). However, it is unlikely that the whole decline in SMI-32-ir neurons would be attributable to cell death. Instead, given the relatively large decline in the level of the protein compared to the decline in the number of immunoreactive neurons we believe that most of the decrease of SMI-32 immunoreactivity is due to the loss of the recognizable epitope caused by phosphorylation. The SMI-32 antibody recognizes a non-phosphorylated site on the carboxyl-terminal regions of the NF-M and NF-H subunits (Soifer et al., 1991). The C-terminal has been documented to undergo extensive phosphorylation during aging (Veeranna et al., 2011) likely as a result of the declining function of phosphatases. It is questionable whether this is a mechanism ensuring increased stability of neurofilaments or a sign of degrading function. Phosphorylation prevents neurofilaments from proteolysis (Goldstein et al., 1987; Pant and Veeranna, 1995) and regulates axonal transport (Shea and Lee, 2011). Moreover, it takes place particularly in axons, stabilizing, and maintaining their caliber, while non-phosphorylated neurofilaments, such as those labeled by SMI-32, remain in somata and dendrites (Sternberger and Sternberger, 1983). On the other hand, an altered neurofilament content is associated with many pathologies. While neurons in Alzheimer’s disease are characterized by the accumulation of phosphorylated neurofilaments in the perikarya (Wang et al., 2001), multiple sclerosis reverses the natural pattern, i.e., dephosphorylation occurs in axons and hyperphosphorylation in perikarya and dendrites (Trapp et al., 1998). Collectively these data provide evidence that altering the phosphorylation status is not uncommon, both in health and disease, and thus suggests that our original SMI-32-ir cells may have been phosphorylated over time, becoming invisible to the labeling in senescence. However, how this specifically affects the processing of auditory signals remains largely unknown. Some insight could be gained by considering the distribution of SMI-32-ir neurons within brain networks and their morphological characteristics.

### The Distribution of SMI-32-ir Neurons and Their Characteristics

Data about the distribution of SMI-32 immuno-reactivity comes mainly from anatomical examinations which utilize its site-specific staining pattern, enabling reliable parcellation of certain parts of the neocortex in cats (Mellott et al., 2010), macaques (Gabernet et al., 1999), rats (Van De Werd and Uylings, 2008), and other mammals, although subcortical examinations are much more sparse (Baizer, 2009; Ouda et al., 2012b). In the study of Ouda et al. (2012b), the authors found that in the rat central auditory system, SMI-32-ir neurons were present in virtually all studied structures although they formed only the minority of neurons (with the exception of medial nucleus of the trapezoid body) with an average content of 10–20%. Such a fraction was also reported in a study focused on the rat neocortex (Kirkcaldie et al., 2002). SMI-32-ir cells are usually larger than the average sized neurons (Campbell and Morrison, 1989; Tsang et al., 2000; Ouda et al., 2012b) with long projecting axons, e.g., motoneurons (Tsang et al., 2000). However, SMI-32 immunoreactivity is better correlated with the degree of myelination, rather than axon length (Kirkcaldie et al., 2002). Thus, SMI-32-ir cells are typically highly myelinated and are consequently fast conductors. In the cortex, they are predominantly type I pyramidal cortical neurons, characterized by burst activity (Molnár and Cheung, 2006). Collectively, these properties strongly suggest that SMI-32-ir neurons belong to the networks involved in processing complex sounds which require fast and time precise conduction of action potentials. These networks can also be seriously affected by the demyelination that accompanies natural aging (Peters, 2009; Tremblay et al., 2012), which would subsequently manifest in symptoms of central presbycusis. We can speculate that demyelination was associated with our decrease in SMI-32 immunoreactivity, but this would require further investigation. Although a direct link between presbycusis and age-related degradation of SMI-32 immunoreactivity is still yet to be revealed, there are physiological alterations indicating deteriorated function of fast conducting neurons that may potentially involve SMI-32-ir neurons. In rodents, these alteration include deteriorated processing of fast FM sweeps (Mendelson and Ricketts, 2001), slowing down of responses to the FM sweeps or increased variability to the same auditory stimuli (Trujillo and Razak, 2013).

In addition to the upper part of the auditory pathway, we evaluated SMI-32 immunoreactivity in the VC which also exhibited a significant age-related decline. Non-phosphorylated neurofilaments have been found in the monkey VC in association with myelin sheaths (Chaudhuri et al., 1996). In mice, myelination of the VC is altered during aging (Tremblay et al., 2012). Combined with our results, these studies suggest that in terms of SMI-32 immunoreactivity the age-related changes observed in the VC parallel those in the AC although the decline is less pronounced. Its functional implications are, however, beyond the scope of this study.

### Age-Related Changes and Comparisons Between the Two Rat Strains

It is a remarkable fact that the aforementioned age-related changes in neuron numbers or protein levels, GAD, calbindin, and calretinin share a mostly similar pattern in both Long Evans and Fischer 344 rats. This is also true for the presented results of SMI-32 immunoreactivity. The two strains differ substantially in age-related pathology of the peripheral auditory system. While Long Evans rats display a slow decline in auditory function, the Fischer 344 strain shows (Syka, 2010) (i) decreasing and eventually disappearing distortion product otoacoustic emissions (Popelar et al., 2006), (ii) as well as degenerative and collagen changes in the stria vascularis (Buckiova et al., 2006, 2007); these pathologies result in increased thresholds of auditory brain responses and qualitatively different responses to startle stimuli and their prepulse inhibition (Rybalko et al., 2012). The inter-strain comparison provides good evidence that age-related changes in the subcortical and cortical auditory structures develop independently (at least in part) of the peripheral condition. Our findings of no observable cell loss in both rat strains thus provide further support for periphery-independent aging of the auditory system.

## Conclusion

In conclusion, we provided unbiased stereological estimates of neuronal numbers in the IC, MGB, and AC. Aging exerts small and non-significant effects on neuron numbers with a decline of about 10% in both examined rat strains. These data suggest that previously found significant declines of markers of inhibitory functions in the rat central auditory pathway, such as GAD (Burianova et al., 2009), parvalbumin (Ouda et al., 2008), and calbindin (Ouda et al., 2012a) cannot be accounted for by significant neuronal loss. Furthermore we report a decline of non-phosphorylated neurofilaments in rats which is presumably due to age related phosphorylation of the SMI-32 recognized epitope. This would interact with the process of myelination and consequently alter the conductance properties of neurons. In the central auditory system, this would seriously affect the processing of complex auditory stimuli. Importantly, age-related changes occur in both Long Evans and Fischer 344 in a similar pattern, suggesting that changes in the central auditory system develop independently of the functional state of the periphery.

## Author Contributions

JB performed western blot evaluation, cell counting, and wrote the article. LO performed perfusions and cell counts analysis. JS coordinated and supervised the study as well as writing the article.

## Conflict of Interest Statement

The authors declare that the research was conducted in the absence of any commercial or financial relationships that could be construed as a potential conflict of interest.
