# Supplementary material for: The influence of aging on the number of neurons and levels of non-phosporylated neurofilament proteins in the central auditory system of rats
Source: Front Aging Neurosci. 2015 Mar 11;7:27. doi: 10.3389/fnagi.2015.00027 (PMC4366680; doi:10.3389/fnagi.2015.00027)
Supplement: Supplementary file 1 [file image_1.pdf]

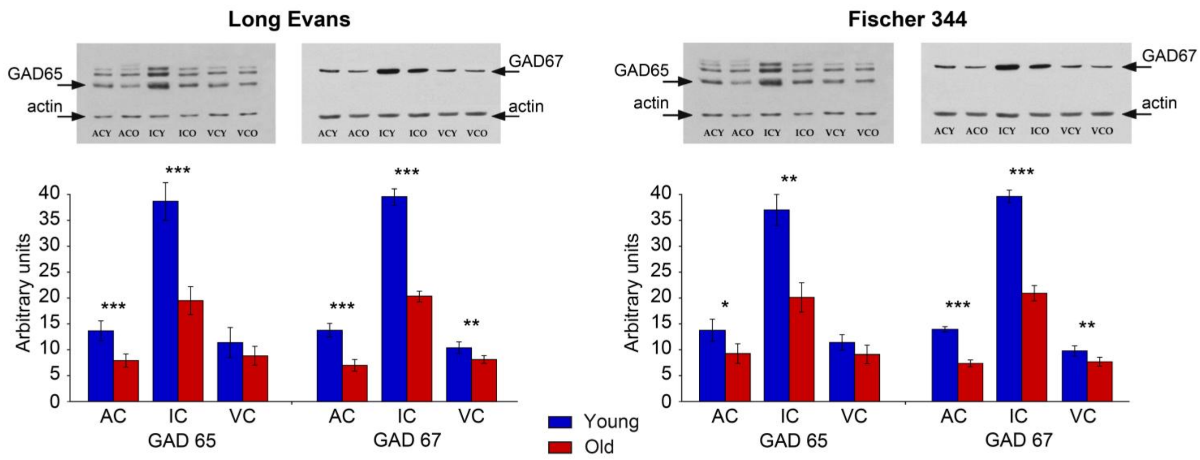


**Supplementary figure 1:** Results of western blot of GAD65 and GAD67 protein analysis in young and old animals of Long Evans and Fischer 344 rats. Corresponding representative films of western blot analysis of GAD65 (65 kDa) and 67 (67 kDa) were developed with the ECL method. Actin, used as an internal control, was detected at the position corresponding to a molecular weight of 42 kDa. Arbitrary units were calculated as the ratio of the optical density of the examined protein and actin (in scanned films analyzed using ImageQuant software). The results presented here are a recalculation of the graphs provided in Figure 4 in the study by Burianova et al. (2009) after adding animals to enhance the statistical power of the data. Current numbers of animals: Long Evans: GAD65 – 8(previously published 6) young, 8(6) old; GAD67 – 10(6) young, 10(6) old; Fischer 344: GAD65 – 4(2) young, 4(2) old; GAD67 – 6(2) young, 6(2) old. For the methodology used in the experiment please refer to Burianova et al. (2009). The abbreviations are: AC – auditory cortex, IC – inferior colliculus, VC – visual cortex, Y – young and O – old animals. The error bars represent S.D. (*- $P<0.05$, **- $P<0.01$, ***- $P<0.001$).
